# Perceived Barriers to Implementing Individual Choosing Wisely^®^ Recommendations in Two National Surveys of Primary Care Providers

**DOI:** 10.1007/s11606-016-3853-5

**Published:** 2016-09-06

**Authors:** Brian J. Zikmund-Fisher, Jeffrey T. Kullgren, Angela Fagerlin, Mandi L. Klamerus, Steven J. Bernstein, Eve A. Kerr

**Affiliations:** 1 Department of Health Behavior and Health Education, University of Michigan, 1415 Washington Heights, Ann Arbor, MI 48109-2029 USA; 2 Division of General Medicine, Department of Internal Medicine, University of Michigan, Ann Arbor, MI USA; 3 Institute for Healthcare Policy and Innovation, University of Michigan, Ann Arbor, MI USA; 4 Center for Bioethics and Social Sciences in Medicine, University of Michigan, Ann Arbor, MI USA; 5 VA Center for Clinical Management Research, VA Ann Arbor Healthcare System, Ann Arbor, MI USA; 6 Department of Population Health Sciences, University of Utah, Salt Lake City, UT USA

## Abstract

**Background:**

While some research has examined general attitudes about efforts to reduce overutilization of services, such as the Choosing Wisely^®^ (CW) initiative, little data exists regarding primary care providers’ attitudes regarding individual recommendations.

**Objective:**

We sought to identify whether particular CW recommendations were perceived by primary care providers as difficult to follow, difficult for patients to accept, or both.

**Design:**

Two national surveys, one by mail to a random sample of 2000 U.S. primary care physicians in November 2013, and the second electronically to a random sample of 2500 VA primary care providers (PCPs) in October–December 2014.

**Participants:**

A total of 603 U.S. primary care physicians and 1173 VA primary care providers. Response rates were 34 and 48 %, respectively.

**Main Measures:**

PCP ratings of whether 12 CW recommendations for screening, testing and treatments applicable to adult primary care were difficult to follow and difficult for patients to accept; and ratings of potential barriers to reducing overutilization.

**Key Results:**

For four recommendations regarding not screening or testing in asymptomatic patients, less than 20 % of PCPs found the CW recommendations difficult to accept (range 7.2–16.6 %) or difficult for patients to follow (12.2–19.3 %). For five recommendations regarding testing or treatment for symptomatic conditions, however, there was both variation in reported difficulty to follow (9.8–32 %) and a high level of reported difficulty for patients to accept (35.7–87.1 %). The most frequently reported barriers to reducing overuse included malpractice concern, patient requests for services, lack of time for shared decision making, and the number of tests recommended by specialists.

**Conclusions:**

While PCPs found many CW recommendations easy to follow, they felt that some, especially those for symptomatic conditions, would be difficult for patients to accept. Overcoming PCPs’ perceptions of patient acceptability will require approaches beyond routine physician education, feedback and financial incentives.

## INTRODUCTION

Nearly three-quarters of U.S. physicians surveyed report prescribing an unnecessary test or procedure at least once per week, and a similar proportion state that patients in their practices request unnecessary tests at least weekly.^1^ Similarly, nearly half of U.S. primary care physicians report that patients are receiving too much medical care.^2^ The Choosing Wisely^®^ (CW) campaign represents a large-scale, evidence-based attempt to address this problem by identifying and raising awareness of low-value health care services that are overused in the U.S.^3^ Since April 2012, the CW campaign has asked national medical specialty societies to each identify five opportunities for reducing overuse of testing, medications, and procedures (ABIM [American Board of Internal Medicine] Foundation; http://choosingwisely.org).^4^ More than 70 societies are now a part of the campaign, and additional specialty groups are continuing to join in this effort.

Although awareness of evidence-based recommendations is a necessary prerequisite for providers to reduce unnecessary use of services, knowledge alone is rarely sufficient to produce dramatic shifts in patterns of clinical practice.^5^^,^ 6 Other provider-, patient-, and organization-specific factors may impede efforts to reduce overuse.^6^^,^ 7 Among clinicians, for example, lack of agreement with specific recommendations, concerns about malpractice,^1^^,^ 8 lack of time to talk with patients,^9^^,^ 10 and the desire to keep patients happy^1^ can pose barriers to reducing the use of some services.

While there are data about providers’ general attitudes towards the CW campaign,^1^ to our knowledge there is no information on providers’ views of individual CW recommendations, or on their perceptions of how difficult specific recommendations would be to implement in clinical practice. Even if providers agree with efforts to reduce overuse of services in principle, concerns about individual recommendations have the potential to impede efforts to reduce use of specific services. In addition, clinicians know that many patients believe that more health care is better than less health care.^11^ As a result, clinicians may find it difficult to deny requests for specific services,^12^ and they could preemptively order tests to avoid eliciting such requests from patients.^6^^,^ 13

The objectives of this study were to (a) measure U.S. primary care providers’ (PCPs) perceptions regarding which CW recommendations applicable to adult primary care were difficult for them to follow, (b) measure those same providers’ perceptions regarding which CW recommendations were difficult for patients to accept, and (c) examine whether the barriers to reducing overuse of low-value services identified by respondents were associated with PCP perceptions of greater difficulty in following specific CW recommendations. We conducted our surveys in two populations of U.S. primary care providers: those practicing primarily in non-federally funded practices, and thus susceptible to private sector rules for financing; and those practicing in the U.S. Department of Veterans Affairs (VA), where all providers are salaried.

## METHODS

The first survey (hereafter, the U.S. Survey) was mailed in November 2013 to the work addresses of a nationally representative random sample of 2000 internal medicine, family medicine, and geriatrics physicians in the United States identified from the AMA Masterfile, which includes almost all doctors of medicine (MD) and doctors of osteopathic medicine (DO). Participants received a $5 incentive in their initial survey packet. Non-respondents received up to two additional surveys over the following 6 months. This design was approved by the University of Michigan’s Health Sciences and Behavioral Sciences Institutional Review Board.

For the second survey (hereafter, the VA Survey), using data from the VA Corporate Data Warehouse, we identified all non-resident primary care providers (physicians, nurse practitioners, and physician assistants) with at least 1 day of direct patient care per week. During October–December 2014, a national random sample of 2500 providers received a pre-notification letter, followed by an email containing a link to an anonymous online survey using REDCap [Research Electronic Data Capture] tools hosted at the VA.^14^ We sent follow-up reminders (maximum of 5) to non-responders by REDCap (email) and UPS overnight mail (surveys included). Respondents were entered into a lottery for a chance to win one of thirty $100 Amazon gift cards. The Ann Arbor VA Human Studies Committee approved this study.

### Survey Instrument Design

We conducted a focused review of all CW recommendations available as of July 2013 to identify those we believed most critically relevant to primary care for adult patients. Recommendations related to pediatrics, obstetrics, or hospital-based care were excluded. Four PCPs (the three physician authors and one other physician) evaluated each of the remaining 84 (out of 135) recommendations for its relevance to primary care for adults. Raters met after the initial ratings, discussed any disagreements, and for each recommendation came to consensus on whether it should be included or not. In particular, raters agreed to include (a) recommendations related to services a PCP might provide or order without active specialty involvement (even if some PCPs might just refer to specialist for an evaluation), (b) recommendations related to services that would be provided whenever a PCP ordered them (i.e., specialty involvement is not required), and (c) recommendations related to a referral that a PCP would initiate. We excluded recommendations for which a PCP would lack sufficient information or expertise to act upon independently.

The raters then graded each recommendation on its likelihood that overuse could cause harm (to either patients or society) and the degree of societal cost (based on prevalence as well as immediate and downstream costs). Differences in ratings were discussed and resolved by consensus. We selected 12 of the set of 41 highest-ranking recommendations (Table 1)—four diagnostic testing, four screening, and four medications—for inclusion in both surveys based on these ratings, eliminating duplicates from different societies.

**Table 1:** The 12 Choosing Wisely^®^ Recommendations Studied

Recommendations related to diagnostic testing
• Don’t do imaging for low back pain within the first 6 weeks unless red flags are present.
• In the evaluation of simple syncope and a normal neurological examination, don’t obtain brain imaging studies (CT or MRI).
• Don’t image for suspected pulmonary embolism (PE) without moderate or high pre-test probability.
• Avoid cardiovascular testing for patients undergoing low-risk surgery.
Recommendations related to screening
• Do not repeat colorectal cancer screening (by any method) for 10 years after a high-quality colonoscopy is negative in average-risk individuals.
• Don’t screen for carotid artery stenosis (CAS) in asymptomatic adult patients.
• Don’t use dual-energy x-ray absorptiometry (DEXA) screening for osteoporosis in women younger than 65 or men younger than 70 with no risk factors.
• Do not repeat colonoscopy for at least 5 years for patients who have one or two small (<1 cm) adenomatous polyps, without high-grade dysplasia, completely removed via a high-quality colonoscopy.
Recommendations related to medications
• Avoid using medications to achieve hemoglobin A1c <7.5 % in most adults age 65 and older; moderate control is generally better.
• Don’t use antimicrobials to treat bacteriuria in older adults unless specific urinary tract symptoms are present.
• Don’t use benzodiazepines or other sedative-hypnotics in older adults as first choice for insomnia, agitation, or delirium.
• Don’t routinely prescribe antibiotics for acute mild-to-moderate sinusitis unless symptoms last for 7 or more days, or symptoms worsen after initial clinical improvement.

The U.S. Survey included a brief description of the Choosing Wisely initiative and a focal section with the 12 selected CW recommendations. For each recommendation, respondents answered two Yes/No questions: (a) “Will it be difficult for YOU to follow this recommendation most of the time?” and (b) “Will it be difficult for MOST PATIENTS to accept this recommendation in practice?” Respondents also (a) rated their overall familiarity with the CW initiative on a five-point scale from “not at all familiar” to “extremely familiar”; (b) answered a set of questions on practice characteristics; and (c) rated eight possible barriers to efforts to reduce overuse of inappropriate services in general, rating each as a major barrier, a minor barrier, or not a barrier.

For the similarly structured VA Survey, the key questions were worded “How easy or difficult will it be for YOU to follow this recommendation most of the time?” and “How easy or difficult will it be for MOST PATIENTS to accept the recommendation in practice?”, with a four-point response scale labeled “very easy,” “somewhat easy,” “somewhat difficult,” and “very difficult.” We collapsed “somewhat” and “very” responses to create a dichotomous variable for our analyses. In addition, we (a) adjusted the practice characteristic questions to reflect relevant categories of clinicians within the Veterans Health Administration, (b) eliminated a question about payment policies that reward ordering more services, and (c) changed the response scale on the question about familiarity with the CW initiative to a three-point scale (“not at all familiar,” “somewhat familiar,” and “very familiar”).

We report the proportion of clinicians in each sample (unadjusted) reporting particular CW recommendations as difficult to follow, difficult for patients to accept, or both. We also report the proportion endorsing particular barriers to reducing overuse. Based on the observed distributions, we then grouped recommendations with similar response patterns. We also examined correlations between PCPs’ reporting of difficulty following each CW recommendation and their ratings of each of the eight general barriers to reducing overuse.

## RESULTS

We received 603 eligible U.S. Survey responses and 1173 eligible VA Survey responses (859 via REDCap and 314 by mail). However, there were also 102 U.S. and 30 VA surveys that were undeliverable/returned to sender. In addition, 123 people returned a U.S. Survey having marked that they did not meet the eligibility criteria, and 33 people returned a VA survey having indicated that they were no longer at the VA, were on deployment or extended leave, or did not provide primary care. Hence, the American Association for Public Opinion Research (AAPOR) RR1 response rates^15^ were 34 % (603/1775) and 48 % (1173/2437), respectively. Demographics and practice characteristics for both survey populations are reported in Table 2. Notably, 67 % of U.S. Survey participants reported that “factors that reflect your own productivity” were a major consideration in determining their compensation, as compared to only 32 % of VA respondents. Conversely, 53 % of VA respondents reported that specific measures of quality of care were a major consideration in determining their compensation, as compared to only 28 % of U.S. Survey respondents. Both surveys included providers highly familiar with the CW initiative, as well as many who were not: 40 % of responses to the U.S. Survey and 63 % of responses to the VA Survey were from clinicians “not at all familiar” with the CW initiative.

**Table 2:** Characteristics of the U.S. Survey and VA Survey Samples

Characteristic	U.S. Survey (*N* = 603) n (%)	VA Survey (*N* = 1173) n (%)
Gender		
Male	397 (66 %)	523 (45 %)
Female	202 (34 %)	630 (55 %)
Transgender	0 (0 %)	2 (0 %)
Completed clinical training		
<10 years	137 (23 %)	257 (22 %)
10–19 years	187 (31 %)	455 (39 %)
20+ years	273 (46 %)	447 (39 %)
Practice arrangement*		
Single physician practice	112 (19 %)	–
Group practice	261 (43 %)	–
Employed by university or teaching institution	53 (9 %)	–
Employed by Veterans Health Administration	22 (4 %)	–
Employed by a managed care organization	41 (7 %)	–
Employed by a hospital	98 (16 %)	–
Employed by other	63 (11 %)	–
Primary compensation for clinical practice		
Billing only	212 (35 %)	–
Salary only	117 (20 %)	–
Salary plus bonus	230 (38 %)	–
Other	37 (8 %)	–
VA practice setting		
VAMC that has residents/clinician trainees	–	380 (33 %)
VAMC that does not have residents/clinician trainees	–	174 (15 %)
CBOC that has residents/clinician trainees	–	58 (5 %)
CBOC that does not have residents/clinician trainees	–	544 (47 %)
Not at all familiar with the Choosing Wisely initiative	228 (40 %)	720 (63 %)

*47 participants marked more than one practice arrangement

*VAMC* Veterans Affairs Medical Center, *CBOC* Community Based Outpatient Clinic

### Attitudes About Specific Choosing Wisely Recommendations

In comparing providers’ perceptions of which CW recommendations were difficult to follow and difficult for patients to accept, three basic patterns emerged: Five of our 12 recommendations appeared both comparatively easy for providers to follow and easy for patients to accept, with <20 % of respondents expressing concern both for themselves and for patients. For three recommendations, a moderate number of providers (between 20 and 40 %) rated these recommendations as both difficult to follow and difficult for patients to accept. For the remaining four recommendations, however, a large number (>40 %) of providers anticipated that patients would find these recommendations difficult to accept.

**Figure 1:**
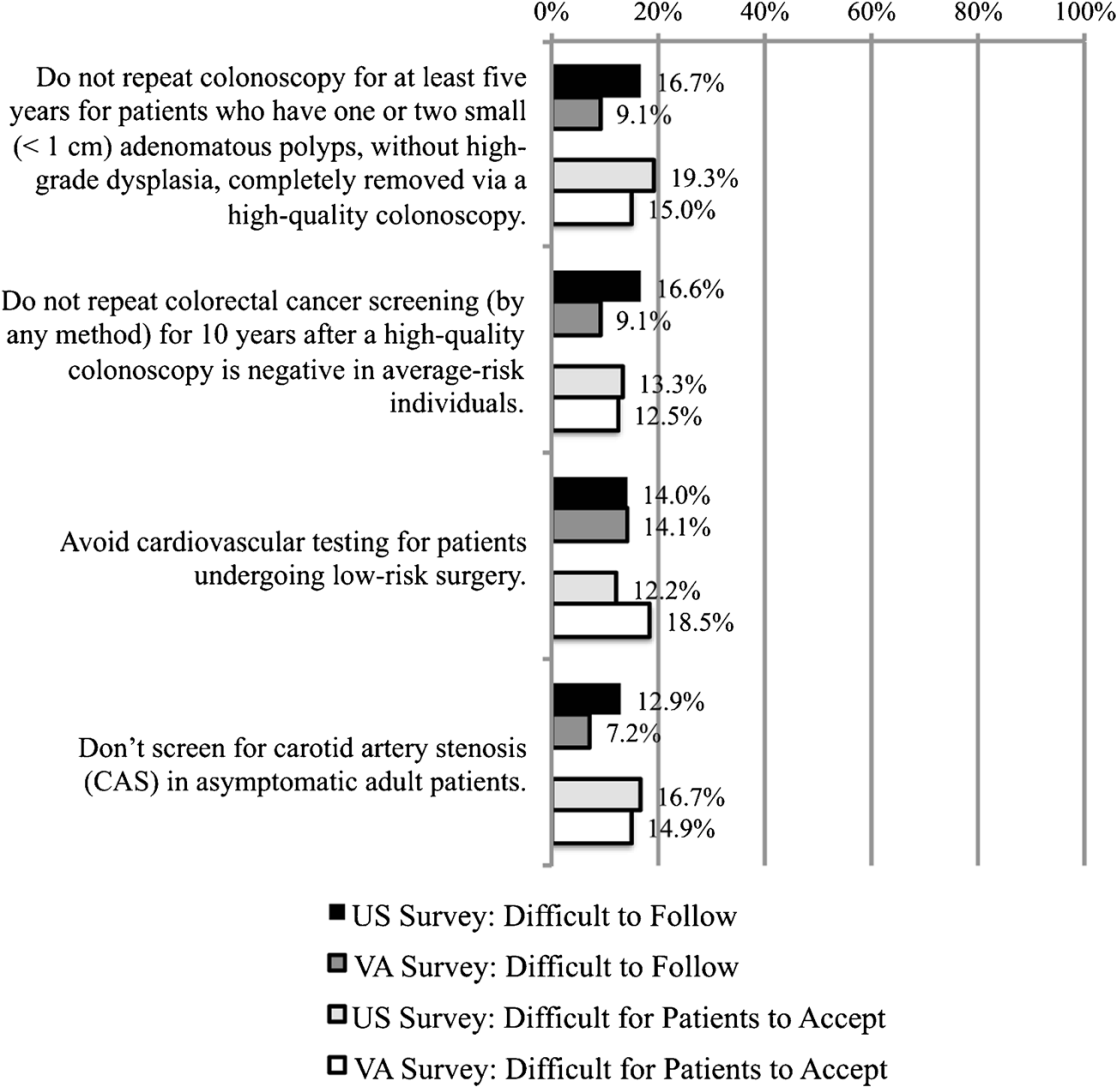
Recommendations that providers believe face few barriers to implementation: Choosing Wisely^®^ recommendations rated by fewer than 20 % of survey respondents as both difficult to follow (*dark bars*) and difficult for patients to accept (*light bars*).

As shown in Figure 1, four recommendations related to not screening or testing asymptomatic patients were perceived by less than 20 % of respondents in both surveys as either difficult to follow or difficult to accept. Specifically, the recommendations (a) to avoid repeat colorectal cancer screening within 5 years if a prior colonoscopy found and removed only 1–2 adenomatous polyps without high-grade dysplasia, (b) to avoid conducting any form of colorectal cancer screening for 10 years if the patient had a negative colonoscopy, (c) to avoid performing cardiovascular testing for patients undergoing low-risk surgery, and (d) to not screen for carotid artery stenosis (CAS) in asymptomatic adult patients were perceived as both comparatively easy to follow and easy for patients to accept.

**Figure 2:**
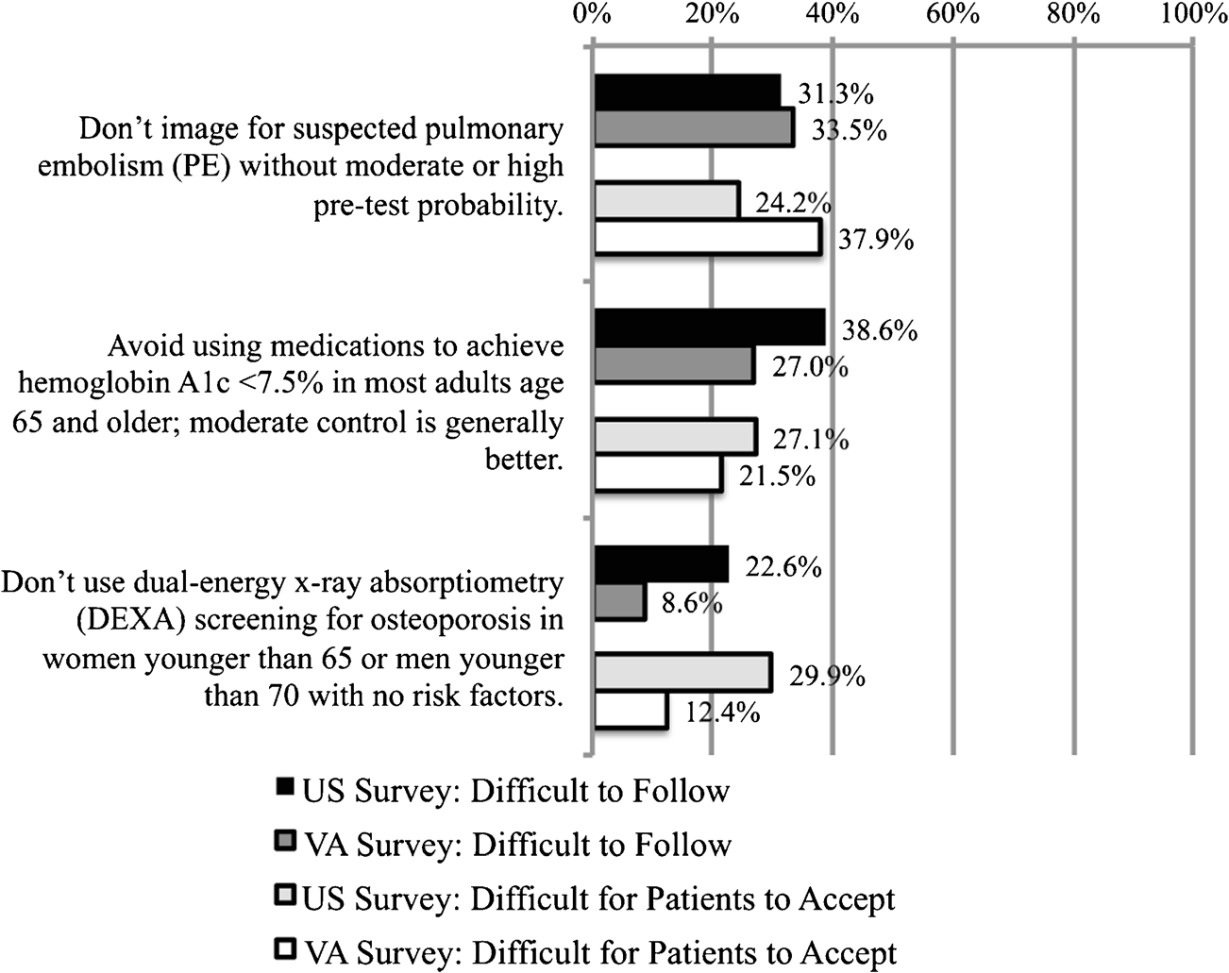
Recommendations that providers believe face moderate barriers to implementation: Choosing Wisely^®^ recommendations rated by between 20 and 40 % of providers as difficult to follow (*dark bars*) and difficult for patients to accept (*light bars*).

Three recommendations evoked greater levels of perceived difficulty within our survey samples (Fig. 2). More than 20 % of respondents in the U.S. Survey rated (a) imaging for suspected pulmonary embolism (PE), (b) avoiding the use of medications to achieve hemoglobin A1c <7.5 % in adults age 65 and older, and (c) avoiding DEXA screening among younger patients with no risk factors as both difficult to follow and difficult for patients to accept. While VA Survey respondents provided similar responses for the first two recommendations, substantially fewer VA survey respondents expressed concern about following the recommendation to limit DEXA screening, even as they agreed that patients would find that recommendation difficult to accept.

**Figure 3:**
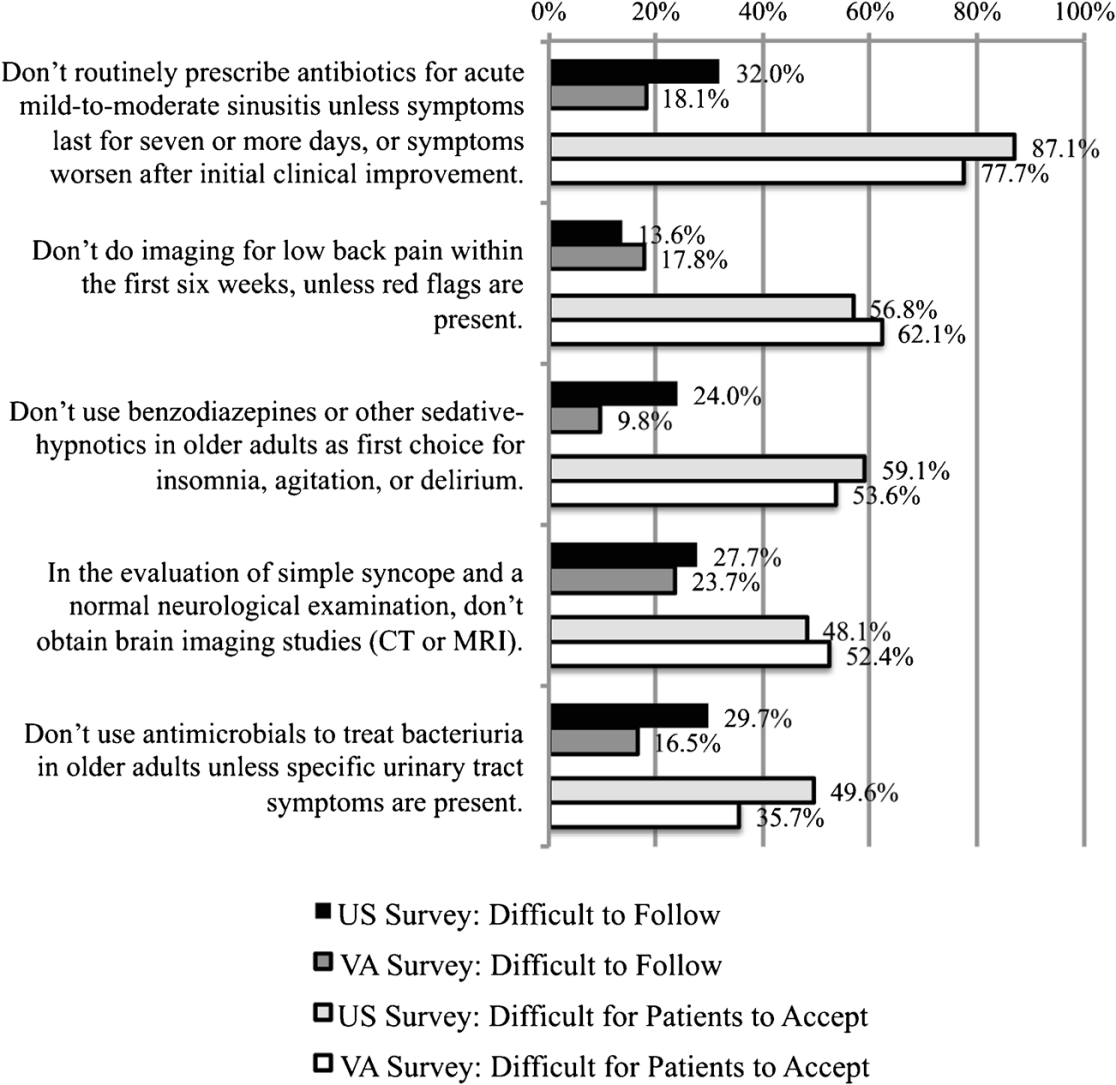
Recommendations that providers believe face major patient acceptance barriers: Choosing Wisely^®^ recommendations rated by more than 40 % of providers as difficult for patients to accept (*light bars*).

A large number (35.7 to 87.1 %) of respondents perceived each of the five remaining recommendations—which are related to medication use and imaging for *symptomatic* conditions—as difficult for most patients to accept (Fig. 3). Specifically, respondents felt that the recommendations (a) to limit use of antibiotics for sinusitis, (b) to avoid imaging for low back pain within the first 6 weeks, and (c) to not use benzodiazepines and other sedative-hypnotics as first choice treatments for insomnia, agitation, or delirium in older adults would be difficult for most patients to accept, even though far fewer respondents felt these recommendations were difficult for providers to follow. Similar patterns were observed for the CW recommendations (a) to avoid brain imaging studies (CT of MRI) in evaluation of simple syncope and (b) to avoid using antimicrobials to treat bacteriuria in older adults unless specific symptoms were present.

### Beliefs About Barriers to Reducing Overutilization of Services

**Figure 4:**
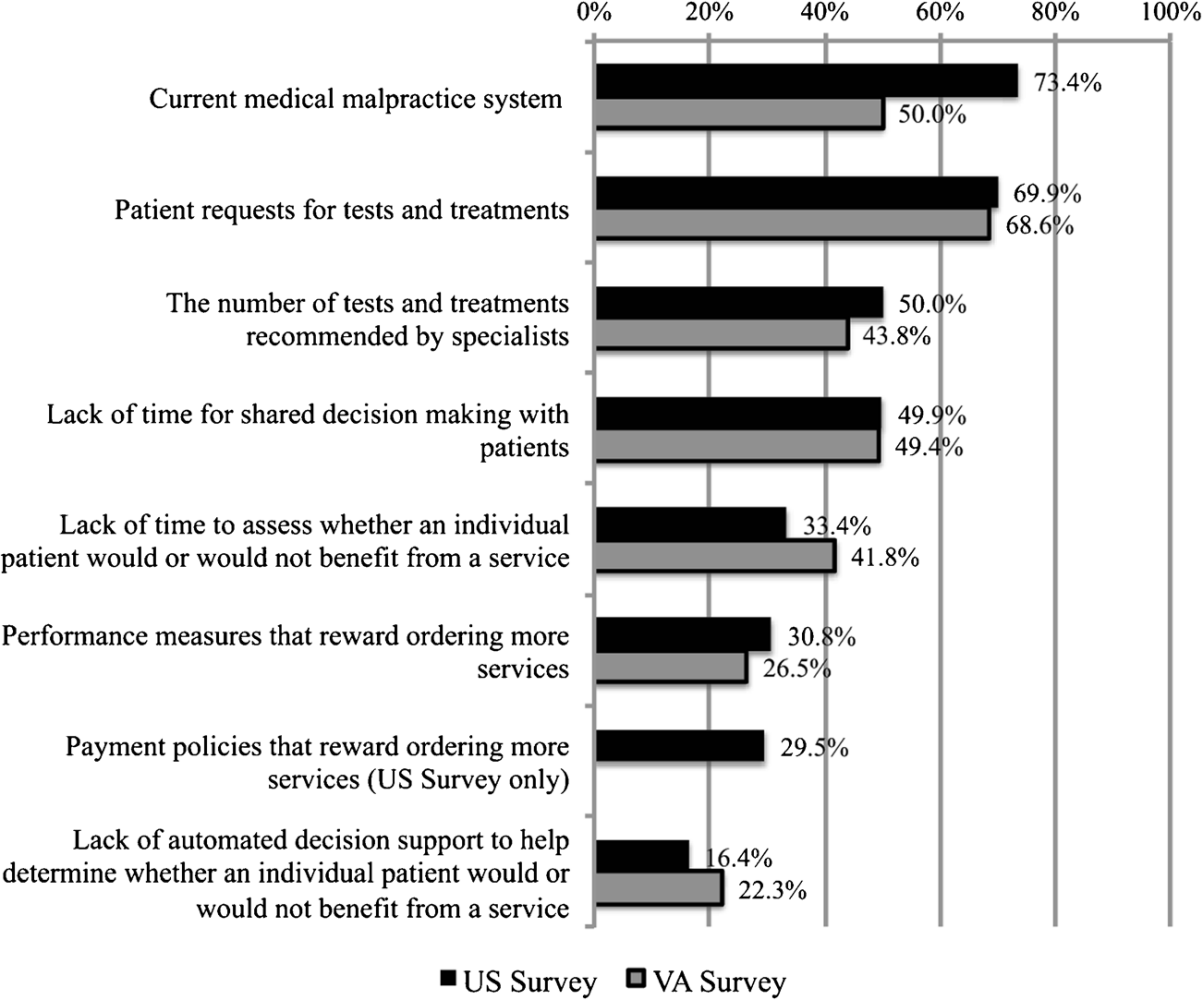
Proportion of survey respondents rating concerns as “a major barrier” to reducing overuse of services.

The perceived prevalence of “major barriers” to efforts to reduce overuse of services (Fig. 4) was remarkably similar across the U.S. and VA Surveys, with “patient requests for tests and treatments,” “the number of tests and treatments recommended by specialists,” and “lack of time for shared decision making with patients” most frequently listed as major barriers. U.S. Survey respondents were more likely than VA Survey respondents to list the malpractice system as a major barrier.

Perceiving patient requests for services as a major barrier was significantly related to marking each of the five recommendations listed in Figure 3 as difficult for patients to accept. For example, 54 % of U.S. Survey providers and 59 % of VA Survey providers who perceived patient requests for services to be a major barrier also believed that patients would find the CW recommendation not to have brain imaging studies for simple syncope difficult to accept. Among providers who did not find patient requests to be a major barrier, those percentages drop to 33 % (U.S. Survey) and 37 % (VA Survey). Perceiving the current medical malpractice system as “a major barrier” to reducing overutilization was associated with perceiving four of the five recommendations as difficult for patients to accept in both surveys. The one exception was the recommendation related to the use of benzodiazepines in older adults.

## DISCUSSION

What will it take to implement Choosing Wisely recommendations on a wide scale? For some recommendations, such as limiting cardiac screening before low-risk surgeries, the answer appears to be “not much,” or perhaps “we’re already doing it.”^16^ Very few survey respondents either perceived difficulty in following that recommendation (and the others shown in Fig. 1) or anticipated that patients would find it difficult to accept. At the same time, however, a majority of those same providers anticipated major challenges in getting patients to accept certain other CW recommendations, such as not prescribing antibiotics for sinusitis and avoiding imaging for lower back pain within the first 6 weeks. In other words, attitudes about one CW recommendation or the CW initiative as a whole cannot be assumed to predict acceptance (or lack thereof) of other recommendations.

The most conspicuous finding of our surveys was the high rates of concern about patient acceptance of the five CW recommendations shown in Figure 3, which all focus on treatment of symptomatic conditions (sinusitis, insomnia/agitation/delirium) or imaging of symptomatic conditions (syncope, low back pain). This pattern is consistent with recent research showing that hospitalists report particularly high rates of overuse of testing for syncope, and attribute this behavior to a desire to reassure patients.^17^

On the one hand, anticipating that patients might have concerns about recommendations related to symptomatic conditions is consistent with literature showing that patients often prioritize care for current symptomatic conditions, whereas clinicians often prioritize screening for and treatment of asymptomatic conditions.^18^^,^ 19 On the other hand, brief communications designed to shape patients’ mental models of their conditions might have a substantial effect on their risk perception,^20^^,^ 21 and hence on whether CW recommendations related to these issues are seen as acceptable. Thus, our findings are concerning, because they suggest that providers might be anticipating patient barriers to acceptance of recommendations to reduce service utilization that either do not exist or could be easily overcome. Unfortunately, such anticipatory concern could become a self-fulfilling prophecy.

We administered the survey to both a national sample of U.S. primary care physicians and a national sample of VA primary care providers in order to represent the opinions of providers practicing in the private sector and in the largest federally funded integrated health care system in the country (serving 8 million veterans). Our less than optimal response rates reflect how difficult it has become to conduct nationally representative surveys of clinicians, especially without large financial incentives.^22^^,^ 23 Response rate concerns also led us to conduct the surveys using different modes (mail and web), which may lead to mode effects on responses. While methodological limitations (different survey modes, timing, and sampling processes) prevent direct statistical comparison of the U.S. and VA surveys, rates of agreement in the two surveys were within a few percentage points of each other on dozens of questions, and the differences that were observed tended to involve VA physicians perceiving certain recommendations (but not others) as easier to follow, which may reflect the VA practice environment. These findings strongly support the universality of concern about implementation challenges facing efforts to reduce overuse. The findings also suggest that such concerns are not primarily driven by reimbursement issues. Yet, as with any survey, there remains the possibility that clinicians with strong opinions about overuse or the CW recommendations might disproportionately have chosen to participate or not participate in our surveys. There is also the possibility that social desirability bias induced respondents to indicate that they were more familiar with the CW initiative or more comfortable with individual recommendations than they actually were. What is clear, however, is that to whatever degree such biases occurred, it was consistent across two independent samples of clinicians operating in very different clinical environments.

The CW campaign has begun to change the discussion about how we practice medicine in the U.S. and abroad.^24^ Yet many providers appear to anticipate patient resistance to CW recommendations for testing or treatment of symptomatic conditions, at least in part related to patients’ requests for such services and litigation concerns. While patients may indeed find such recommendations particularly difficult to accept, it is also possible that providers’ beliefs about patient concerns may be inaccurate and that patients’ attitudes may change with effective communication. Anticipation of patient concerns should not be allowed to create undue hesitation in efforts to implement such initiatives. However, it is likely that interventions will need to extend beyond PCP-directed education, feedback, and incentives, in order to impact change for recommendations that PCPs fear patients will reject. Indeed, the cross-recommendation variations in provider attitudes that we document imply that implementation efforts will need to be tailored to the specific barriers in implementing each CW recommendation. Such tailoring may be critical in producing meaningful reductions in overuse of low-value health care services.
